# Maternal Immunization Affects *In Utero* Programming of Insulin Resistance and Type 2 Diabetes

**DOI:** 10.1371/journal.pone.0045361

**Published:** 2012-09-18

**Authors:** Claudia Eberle, Esther Merki, Tomoya Yamashita, Susie Johnson, Aaron M. Armando, Oswald Quehenberger, Claudio Napoli, Wulf Palinski

**Affiliations:** 1 Department of Medicine, University of California San Diego, La Jolla, California, United States of America; 2 Department of Internal Medicine, Kobe University School of Medicine, Kobe, Japan; 3 Department of Pharmacology, University of California San Diego, La Jolla, California, United States of America; 4 Department of General Pathology and Excellence Research Center on Cardiovascular Disease, First School of Medicine, Second University of Naples, Naples, Italy; Otto-von-Guericke University Magdeburg, Germany

## Abstract

Maternal immunization with oxidized lipoproteins prior to pregnancy protects against atherogenic *in utero* programming by gestational hypercholesterolemia and enhances beneficial lymphocyte-dependent immune responses in offspring. To determine whether *in utero* programming and immunomodulation also affect insulin resistance (IR) and type 2 diabetes, we investigated the effects of immunization on glucose and insulin responses in LDL receptor-deficient mice fed regular or 60% sucrose diets, as well as in offspring fed 0.5% cholesterol or 60% sucrose diets. IR was assessed by fasting glucose and insulin levels, oral glucose tolerance tests, glucose clamps, pancreatic immunohistochemistry and plasma free fatty acid concentrations. Immunizations improved glucose responses in both genders and protected both immunized mice and their offspring against IR and type 2 diabetes. Protection occurred even under euglycemic conditions, but was greatest in obese males exposed to very obesogenic/diabetogenic conditions. Hyperinsulinemic euglycemic clamps confirmed that maternal immunization protected mainly by reducing IR, but pancreatic immunocytochemistry also indicated some protection against beta cell damage. Maternal immunization was associated with marked regulation in offspring of 4 genes relevant to diabetes and 19 genes of importance for oxidative stress, as well as increased hepatic activities of key antioxidant enzymes. These findings establish that targeted immunomodulation may be used to protect immunized subjects and their offspring against IR and type 2 diabetes, and thus to reduce cardiovascular risk. They also support the notion that *in utero* programming influences offspring disease not by a single mechanism, but by multiple systemic effects.

## Introduction

Extensive epidemiological evidence links the *in utero* environment with atherosclerosis and diabetes later in life. The increasing prevalence of maternal obesity and related dysmetabolic conditions is therefore expected to lead to a wave of cardiovascular disease in their children, but little is known about the mechanisms of pathogenic *in utero* programming and its prevention. However, studies on one such maternal dysmetabolic condition, hypercholesterolemia, have shown that interventions in mothers may provide life-long benefits to offspring [1], [2]. Maternal hypercholesterolemia during pregnancy, even if only temporary, is associated with increased fatty streak formation in human fetal arteries and accelerated atherogenesis during normocholesterolemic childhood [3]–[5]. Although an influence of genetic differences could not be ruled out in humans, conclusive evidence for atherogenic programming by maternal hypercholesterolemia was obtained in animal models [1], [6], [7]. The recent observation that offspring with heterogeneous familial hypercholesterolemia (FH) who inherited FH from their mother and were therefore exposed to higher maternal cholesterol levels *in utero* had greater overall mortality than those who inherited FH from their father also supports the pathogenic role of maternal hypercholesterolemia [8].

Some of the mechanisms by which maternal hypercholesterolemia affects developmental programming have already been established, for example the increased oxidative stress associated with elevated cholesterol levels [1], [6], [7]. The mechanisms of transplacental cholesterol transfer from mother to fetus have been elucidated [9]–[11]. A number of potential mechanisms by which fetal programming may enhance atherogenesis have also been identified. For example, maternal hypercholesterolemia leads to endothelial dysfunction and impaired vascular relaxation [12], [13], altered cholesterol synthesis [14], altered arterial gene expression [15], and increased plasma levels of proinflammatory eicosanoids [16]. The observation of impaired glucose homeostasis in offspring of rats fed high-fat diet throughout pregnancy and lactation suggested that maternal hypercholesterolemia and oxidative stress may also affect *in utero* programming of IR [17].

From a translational perspective, the most important aspect of *in utero* programming is the possibility of achieving long-term protection of offspring by brief interventions in mothers. Cholesterol-lowering by cholestyramine or antioxidant treatment during pregnancy markedly reduces postnatal atherogenesis in rabbits and mice [1], [2], [6]. Maternal immunization with oxidized LDL (OxLDL) prior to hypercholesterolemic pregnancy also protects offspring [2]. This may not be due exclusively to the prevention of atherogenic programming by maternal hypercholesterolemia and oxidative stress, but may also involve enhanced postnatal immune responses to atherogenic antigens. In fact, even though fetal lymphocytes are immature and incapable of adaptive responses, maternal OxLDL immunization programs antigen-specific B cell-dependent IgM immune responses in offspring [2]. We therefore postulated that modulation of *in utero* immune programming would not only reduce atherosclerosis, but also the susceptibility to other conditions influenced by oxidative stress, immune mechanisms and inflammatory processes, including IR and type 2 diabetes.

We now provide evidence that maternal immunomodulation indeed affects *in utero* programming of IR and that immunization with oxidized lipoproteins protects immunized murine mothers and their offspring against IR and diabetes under euglycemic, hypercholesterolemic, and hyperglycemic conditions. For this purpose, we investigated the effects of immunization with LDL oxidized to various extent on glucose and insulin metabolism. This was done both in immunized subjects, because altered glucose metabolism in immunized mothers may influence *in utero* programming, and in offspring, to establish the effects of *in utero* programming on IR and type 2 diabetes. Given that moderate protective effects may be overwhelmed by harsh diabetogenic conditions, offspring were exposed to either a moderately obesogenic diet consisting of standard chow enriched with 0.5% cholesterol or an obesogenic diet containing 60% sucrose. Finally, effects on IR in males and females were investigated separately, because the antiatherogenic effects of maternal immunization are greater in male mice than in females [2].

## Methods

### Experimental Design

**Figure 1:**
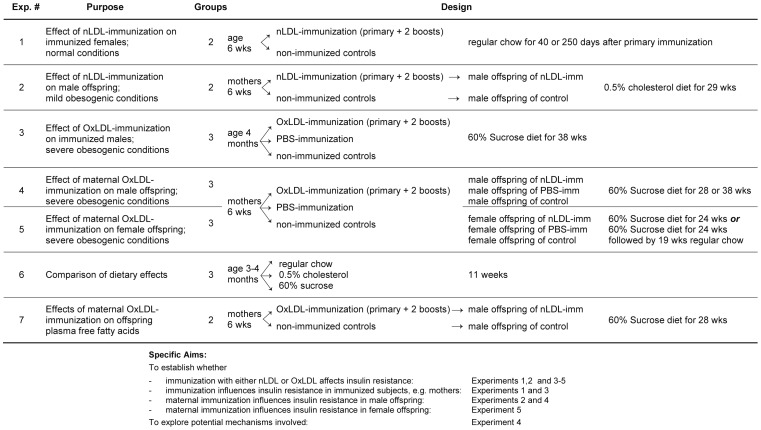
Overview of the experimental design and aims.

Experiments were carried out in 365 LDL receptor-deficient (LDLR^−/−^) mice from our colony, established from founders bred back into the C57BL/6 background for 10 generations. To determine the effects of immunization in immunized subjects and in offspring of both genders under different dietary conditions, seven experiments were carried out. Their purpose and experimental design is outlined in **Figure 1**. Experiment 1 investigated the effects of immunization with naturally oxidized LDL (nLDL) in 24 immunized females and 25 non-immunized controls. Immunization (primary and 2 boosts) began at age 6 weeks and oral glucose tolerance tests (OGTTs) were performed 40 or 250 days thereafter. Experiment #2 tested the effect of maternal nLDL immunization in male offspring of 24 nLDL-immunized and control mothers fed regular chow and bred after completion of immunization. From their offspring, two groups of male offspring roughly matched for number, birth dates and body weight were assembled (total n = 26). Upon weaning at 4 weeks, offspring were fed regular chow supplemented with 0.5% cholesterol (TD97234, Harlan-Teklad), and OGTTs performed after 29 weeks. Experiment 3 investigated the direct effect of immunization with extensively oxidized LDL (OxLDL) under severely obesogenic and diabetogenic conditions in 22 males of the parental generation. In contrast to the first two experiments, this and the following experiments included an additional control group immunized with PBS. Immunized males, age 4 months, were fed a high-carbohydrate, low-fat diet termed 60% Sucrose (TD 05516, Harlan Teklad, containing 60% sucrose, 20.7% casein, 7.696% cellulose, 5% lard, 5% mineral mix, 1% vitamin mix, 0.3% DL-methionine, 0.3% choline bitartrate, 0.004% zinc carbonate) for 38 weeks. Experiments 4 and 5 investigated the effects of maternal OxLDL immunization on 65 male and 60 female offspring, respectively, exposed to severely diabetogenic conditions. In order to ensure that sufficiently large offspring groups matched for age, gender, and weight would be generated, 54 mothers fed regular chow were immunized with OxLDL or PBS, or served as untreated controls. Their male offspring were fed the 60% Sucrose diet for 28 or 38 weeks, beginning at weaning (n = 39 and 16, respectively). Female offspring were fed the same diet for 24 or 36 weeks (n = 54 and 16, respectively). Effects on glucose and insulin responses in Experiments 1–5 were assessed by OGTTs, hyperinsulinemic euglycemic clamps, pancreatic immunohistochemistry, qRT-PCR of mRNA expression in hepatic and cardiac tissues, and measurement of antioxidant enzyme activities. Differences in the length of dietary exposure in experiments 4 and 5 were mainly due to our logistical inability to perform all measurements at the same time, in particular clamps requiring surgeries and rate-limiting equipment. Experiment 6 compared the effects of the three experimental diets (regular chow, 0.5% cholesterol, 60% sucrose) in 31 males after 11 weeks. Experiment 7 compared free fatty acids in 32 offspring of OxLDL-immunized and control mothers fed 60% sucrose for 24 weeks, as well as 11 control mice fed regular chow (maternal n = 9).

### Ethics Statement

All experiments were performed under protocol S02010 approved by the Institutional Animal Care and Use Committee of the University of California, San Diego.

### Immunizations

Mice were immunized with homologous nLDL, OxLDL, or PBS. LDL was isolated by density gradient centrifugation [18]. nLDL was generated from LDLR^−/−^ mice fed a 1.25% cholesterol and 21% milk fat diet (TD96121, Harlan Teklad) [18]. LDL tested for endotoxin levels by chromogenic Limulus amoebocyte assay (QCL-1000; BioWhittaker, Wakersville, MD) contained less than 2 ng lipopolysaccharides/mg protein. To generate naturally oxidized (nLDL), LDL was then stored without antioxidant protection at 4°C for about 3 weeks before use. OxLDL consisted of an equal mixture of malondialdehyde-modified and copper-oxidized LDL generated as previously described [2], [18], to induce antibodies to a broad spectrum of oxidation-specific epitopes. Primary immunization consisted of inguinal injection of the antigen (60 µg protein/kg BW) emulsified with an equal amount of complete Freund’s Adjuvant (FA) (Sigma-Aldrich, St. Louis, MO); 2–3 boosts in biweekly intervals were carried out with 30 µg antigen in incomplete FA (Sigma-Aldrich).

### Oral Glucose Tolerance Tests

Blood (20–25 µl) was collected from the retro-orbital plexus of 6 hour- fasted mice briefly anesthetized with isofluorane, using a precision vaporizer, prior to glucose gavage and 15, 30, 60, 90, and 120 min thereafter. Gavage consisted of intragastric administration of 1.5 g glucose/kg BW. Glucose was determined by Ascensia Contour glucometer and blood glucose test strips (Bayer Health Care, Mishawaka, IN), and plasma insulin levels by Mercodia Ultrasensitive Mouse Insulin Kits (American Laboratory Products, Windham, NH).

### Hyperinsulinemic Euglycemic Clamps

Mice were anesthetized with isofluorane (Anesthesia System V-10, Vet-Equip, Pleasanton, CA) and a saline-filled 25-gauge catheter was implanted under sterile conditions in the right jugular vein, using a Leica MZ6 stereo microscope (Leica Microsystems, Buffalo Grove, IL). Catheters were exteriorized through the dorsal neck. Incision sites were treated with lidocaine and sealed with surgical glue. Body temperature was maintained throughout surgery by a heated circulating water blanket. Animals were then kept on a swivel tether system (Instech Solomon, Plymouth Meeting, PA) with free access to food and water for 48 hours, during which the catheter was perfused with 40 µl/hr saline with a PHD 2000 perfusor pump (Harvard Apparatus, Holliston, MA). After careful consideration of the various conditions of clamps in the literature [19], we opted for a less stressful fasting period of 5 hours, a high constant infusion rate of insulin (12 mU/kg/min; 1 µl/min) (Humulin, Ely Lilly, Indianapolis, IN) without priming, and a variable infusion rate of 50% dextrose for up to 2 hours, both via a Dual Perfusor Pump 33 (Harvard Apparatus) [20]. Blood glucose levels were determined in 15 min intervals until a steady state of dextrose infusion and euglycemia (100–150 mg/dl) was maintained over 30 min.

### Lipid Analysis

The cholesterol content of lipoprotein fractions in the plasma was determined by fast-performance liquid chromatography (FPLC) [21]. Free fatty acids were extracted and analyzed by gas chromatography mass spectrometry (GC/MS) essentially as described [22]. Briefly, 100 µl of plasma was supplemented with deuterated internal standards (Cayman Chemical, Ann Arbor, MI) and extracted twice with 0.05 N methanolic HCl/isooctane (1∶3, v/v) and the combined isooctane layers were evaporated to dryness. The extracted free fatty acids were derivatized with pentafluorobenzyl bromide and the fatty acid esters were analyzed by GC/MS on an Agilent 6890N gas chromatograph equipped with an Agilent 5973 mass selective detector (Agilent, Santa Clara, CA). Fatty acid quantitation was achieved by the stable isotope dilution method.

### mRNA Expression

Approximately 30 mg of liver tissue or 20 mg of cardiac ventricle tissue were dissected and placed into RNAlater (Qiagen, Valencia, CA). RNA was isolated using NucleoSpin mini RNA isolation kits (#740955, Macherey-Nagel, Bethlehem, PA) according to the standard protocol. Preparation of ventricular tissue included an extra proteinase K digestion step. RNA was quantitated using a spectrophotometer (NanoDrop Technologies, Wilmington, DE).

We first performed conventional qPCR to determine suitable normalization genes. For this purpose, 1 µg of total RNA was reverse transcribed, using the Clontech Sprint RT complete double primed kit (#PT4048-2) according to the protocol. The resulting cDNA was diluted 1∶40 (liver) or 1∶8 (ventricle). Quantitative PCR was performed with a Rotor-Gene 3000 PCR (Corbett Research, Sydney, Australia), using LUX Primers (Invitrogen, Carlsbad, CA). Cycling conditions were: 1 cycle of 95°C for 2 min; 50 cycles of denaturation 95°C for 5 sec, hybridization/elongation 60°C for 20 sec, followed by a melt curve analysis ranging from 60°C to 95°C, ramp time 1°C per step, waiting for 60 sec for the first step and 5 sec for all subsequent steps. The PCR mix contained 1x JumpStart Taq Ready Mix (# P2893, Sigma-Aldrich) and 5 µl of diluted cDNA in a total volume of 25 µl per reaction. Normalization genes were selected from 12 candidates using GeNorm kit for mouse perfect probe (# ge PP-12-mouse) and qBase plus software (www.primerdesign.eu). Selected probes were ACTB (beta actin cytoplasmic) and RPL13a (ribosomal protein L13a).

Hepatic expression of 84 genes related to oxidative stress and 84 genes of interest for diabetes was then assessed with the Oxidative Stress and Antioxidant Defense qPCR Array (PAMM-065) and the Diabetes qPCR Array (PAMM-023), respectively (SA Biosciences/Qiagen). Reverse transcription was performed using 1 µg of total RNA and the RT^2^ First Strand cDNA synthesis kit #330401 (Qiagen) containing RNA quality and genomic DNA contamination controls. qPCR was performed on a Roche Lightcycler 480 using the corresponding RT^2^ SYBR green Master Mix #330502 (Qiagen). cDNA synthesis and qPCR were performed according to manufacturer’s protocols. Array data were analyzed with the manufacturer’s online data analysis software (http://www.sabiosciences.com/pcrarraydataanalysis.php), using the same normalizing genes we had previously chosen with GeNorm.

### Antioxidant Enzyme Activities

Approximately 50 mg liver and 60 mg ventricle tissue were flash frozen in liquid nitrogen and stored at −80°C until processing. Tissues were placed in 500 µl ice cold PBS containing 1 mM EDTA, homogenized with a tissue homogenizer (Omni International) and transferred into an Eppendorf tube containing another 500 µl ice cold PBS with 1 mM EDTA. The homogenate was centrifuged for 5 min at 1500×g at 4°C, and an aliquot of the supernatant was removed for measurement of total superoxide dismutase (SOD) activity. The remaining homogenate was then centrifuged at 10,000×g for 15 min and the supernatant was aliquoted for determination of cytosolic SOD, glutathione peroxidase (GPx), and catalase (CAT) activities. The pellet was resuspended in 300 µl of SOD sample buffer and again homogenized for mitochondrial SOD measurement. Samples from all mice were stored at −80°C for joint evaluation in the same assay. Activities were determined with Superoxide Dismutase, Catalase, and Glutathione Peroxidase Assay kits (#706002, #707002, and #703102; Cayman Chemical, Ann Arbor, MI) according to the manufacturer’s protocols, and absorptions measured with a Synergy 2 Multi-Detection Microplate Reader (BioTek, Winooski, VT). Protein concentrations were determined by BCA Protein Assay (#23225, Pierce Biotechnology, Rockford, IL).

### Immunohistochemistry

The pancreas was carefully dissected under a Leica MZ6 stereo microscope (Leica Microsystems, Buffalo Grove, IL), paraformaldehyde-fixed, and paraffin-embedded for immunohistochemistry. Sections (6 µm thick) were immunostained with 1∶1000 dilutions of mouse monoclonal antibodies against glucagon or insulin (G2654 and 12018, respectively, Sigma-Aldrich, St. Louis, MO), using a Mouse-on-Mouse kit (BMK-2202,Vector Laboratories, Burlingame, CA) and an avidin-biotin-alkaline phosphatase detection system with a red color substrate (Vectastain ABC-AP kit AK-5000, Vector Red SK-5100, Vector Laboratories). Sections stained without primary antibodies served as controls. Glucagon-positive pancreatic alpha cells and insulin-positive beta cells were visually identified and counted. Results were expressed as cells per section or beta cell/alpha cell ratios.

### Statistics

Normal-distributed data were compared by Student’s unpaired two-tailed T-test and differences considered significant when P≤0.05.

## Results

### Dietary Effects

**Figure 2:**
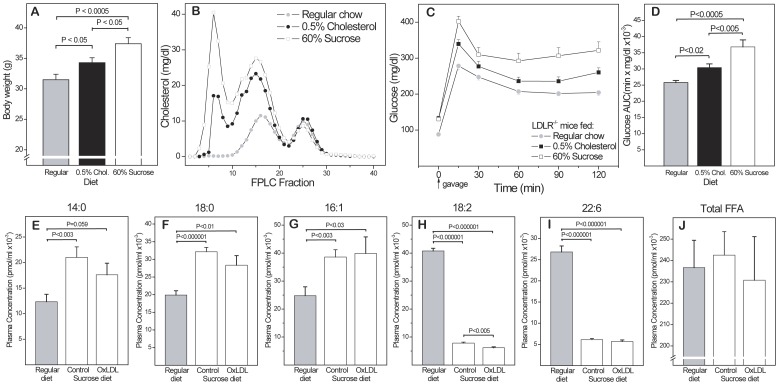
Characterization of experimental diets. (**A**) Effects of regular chow, 0.5% cholesterol diet and 60% sucrose diet on (**A**) body weight, (**B**) lipoprotein profiles and (**C,D**) glucose response in male mice after 78 days on diet (n = 31). FPLC analysis was performed on pooled plasma samples from 2–3 mice per group. Glucose responses were assessed by OGTT) on 31 mice (C) and differences in the area under the curve (AUC) determined by unpaired T-test (D). (**E–I**) Plasma concentrations of major free fatty acids (FFAs) in male offspring of OxLDL-immunized (OxLDL) and non-immunized mothers (controls) after 24 weeks on 60% sucrose or regular diet (n = 32). (**J**) Total FFAs.

The effects of the diets administered are compared in **Figure 2**. Cholesterol levels increased over time in all groups, reaching a plateau after 3–4 months (346±13 mg/dl in the control group, 889±25 mg/dl in the 0.5% cholesterol group and 1245±61 mg/dl in the 60% sucrose group). As expected, the 0.5% cholesterol diet enhanced the spontaneous hypercholesterolemia of LDLR^-/-^ mice, but the 60% sucrose diet was more obesogenic and affected lipoprotein profiles and OGTT glucose responses to a greater extent, especially in males **(A–D)**. The effect of this diet on plasma concentrations of 33 free fatty acids (FFAs) were assessed in males fed 60% sucrose or regular diet for 24 weeks. Representative FFAs with the highest concentrations are shown in **(E–I)**. Most saturated and monounsaturated FFAs followed the patterns shown for 14∶0, 18∶0 and 16∶1, i.e. were greatly increased by the sucrose diet. Maternal OxLDL immunization tended to reduce this increase. This was statistically significant for some FFAs not shown, e.g. 20∶0 and 22∶1 (P<0.01 for both), but did not come close to compensating for the effect of the sucrose diet. In contrast, many polyunsaturated FFAs were decreased by the sucrose diet, again with only marginal influence of maternal immunization (H,I). As a result of these opposing trends, total FFAs were not significantly affected by the sucrose diet, and maternal immunization showed only a slight trend towards lowering them **(J)**.

### Immunization with nLDL Protects Immunized Euglycemic Females

**Figure 3:**
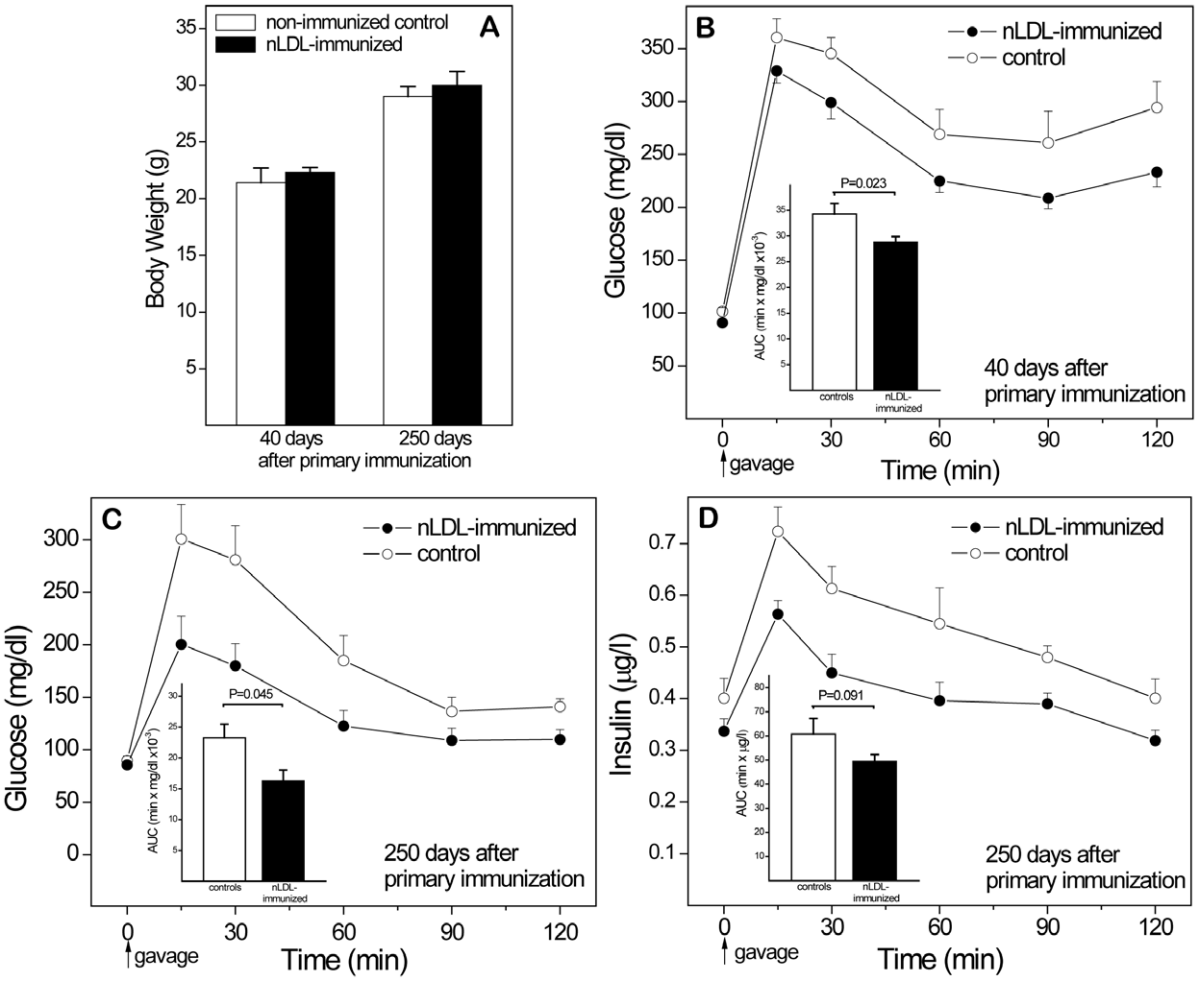
Protective effects of nLDL immunization in euglycemic female mice fed regular chow. To assess direct effects of immunization on glucose responses, OGTTs were performed on separate groups of female mice 40 and 250 days after the primary immunization. Controls were non-immunized. (**A**) Body weights at both time points. (**B**) Glucose responses after 40 days. (**C,D**) Glucose and insulin responses after 250 days. AUC, Area under the curve. n = 24 (40 days) and 25 (250 days).

Immunization with either nLDL or OxLDL reduces atherogenesis in immunized animals [23]–[27]. Because immunization of mothers may affect fetal programming by influencing maternal glucose and insulin, we first investigated the consequences of nLDL immunization in females under normal dietary conditions. Glucose and insulin responses were determined 40 days after primary immunization (corresponding to the time shortly before pregnancy in mothers of experiments 3, 5, and 6), and 250 days after immunization **(** **Figure 3** **)**. All mice gained weight over time, but immunization had no effect on body weights **(A)**. Better glucose responses in immunized subjects were first noted at day 40 **(B)**, and became more evident 250 days after primary immunization **(C)**. Insulin measurements showed a clear trend towards higher levels than in nLDL-immunized mice **(D)**. The observation of higher glucose levels despite greater insulin release suggests that older control females develop some degree of IR even on regular chow, and that nLDL-immunization is protective even under conditions of euglycemia (and moderate spontaneous hypercholesterolemia).

### Maternal Immunization with nLDL Protects Male Offspring under Mildly Obesogenic Conditions

**Figure 4:**
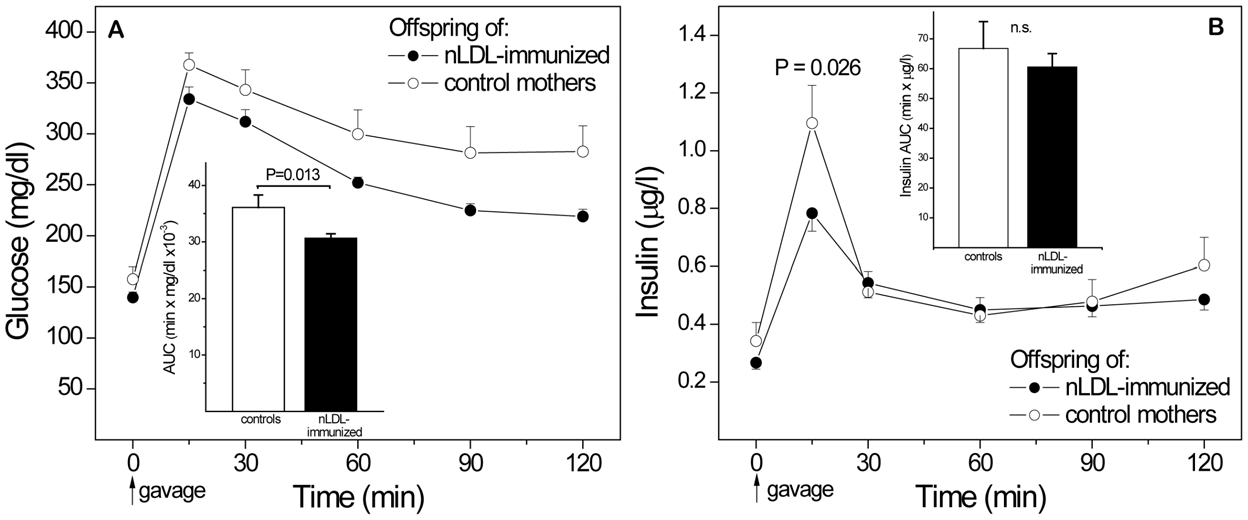
Protective effects of maternal immunization with naturally oxidized LDL (nLDL) on male offspring fed 0.5% cholesterol diet. (A) Glucose response in an OGTT in offspring after 29 weeks on diet. (B) Insulin responses in the same OGTT. AUCs for glucose and insulin are shown as insets; statistical significances for individual time points are not indicated. n = 26.

To explore the effects of maternal immunization on offspring glucose and insulin metabolism under mildly obesogenic conditions, male offspring of nLDL-immunized mothers and non-immunized controls were fed the 0.5% cholesterol diet for 29 weeks, starting at weaning **(** **Figure 4** **)**. Time-averaged plasma cholesterol levels and terminal body weights were similar (data not shown). An OGTT performed at the end of dietary intervention showed better glucose responses in offspring of nLDL-immunized mothers **(A)**. Fasting glucose was similar in both groups, but glucose at most time points after gavage and the area under the curve (AUC, inset) was significantly lower in offspring of immunized mothers, compared to offspring of controls, even though OGTT insulin peaks were higher in the control group, consistent with greater compensatory hyperinsulinemic responses and greater IR in controls **(B)**.

### Immunization with OxLDL Protects Immunized Males Against IR under Highly Obesogenic Conditions

**Figure 5:**
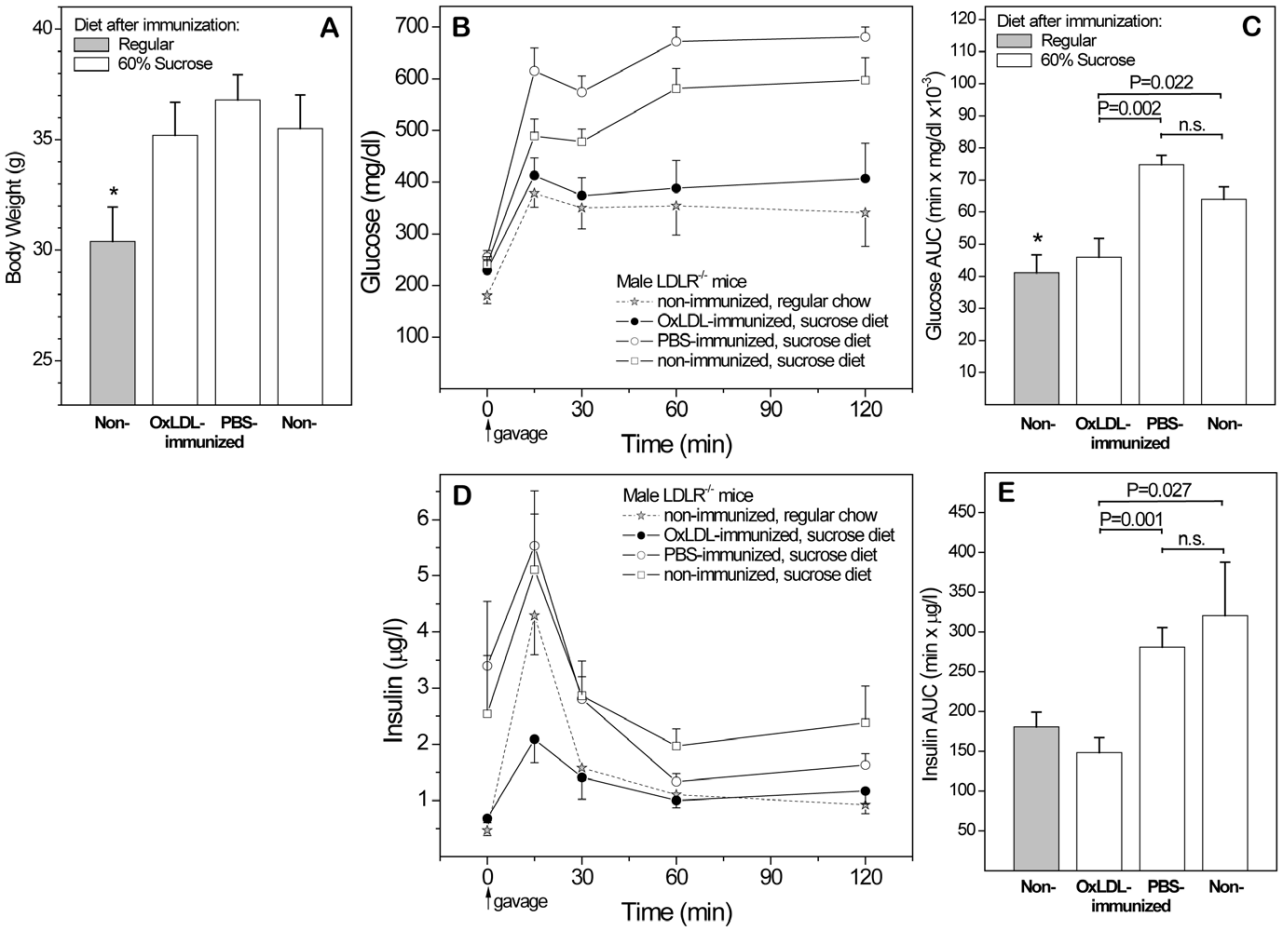
Protective effects of OxLDL immunization in male mice fed 60% sucrose. To assess direct effects of immunization with extensively oxidized LDL in immunized mice exposed to more obesogenic conditions, 4 month old male LDLR^-/-^ mice immunized with OxLDL or PBS and controls were fed 60% sucrose for 38 weeks. Age-matched non-immunized mice fed regular chow served as additional control. (**A**) Body weights. (**B,C**) OGTT glucose response curves and AUCs. (**D,E**) Corresponding OGTT insulin curves and AUCs. n = 22.

Having established that nLDL exerts beneficial effects on glucose and insulin responses in immunized females and their offspring under mildly obesogenic conditions, we then investigated whether OxLDL protects immunized mice under highly obesogenic conditions. For this and all subsequent experiments, we included an additional control group immunized with PBS (with FA). Based on previous atherosclerosis studies, we anticipated that this would yield intermediate results, due to the presence of oxidation-specific epitopes in the mycobacterial adjuvant and the generation of additional oxidative neoepitopes by the local inflammation caused by FA. We first tested the direct effect of OxLDL-immunization in older males immunized at age 4 months and fed the 60% sucrose diet for 30 weeks **(** **Figure 5** **)**. Compared to non-immunized controls fed regular chow, all three groups on sucrose diet gained substantial weight **(A)**. An OGTT at the end of the dietary intervention showed significantly better glucose responses in OxLDL-immunized males than in PBS-immunized and non-immunized controls, similar to those of normal non-obese mice fed regular chow **(B,C)**. Fasting insulin and insulin responses after gavage were significantly lower in OxLDL-immunized mice than in both sucrose fed controls **(D,E)**. Again, the higher glucose levels concomitant with greater compensatory insulin responses in the control groups are indicative of IR, and OxLDL immunization protected against the onset of IR induced by this extreme diet. Together, results of Figs. 3 and 5 show that immunization with both naturally and extensively oxidized LDL protect immunized subjects under a variety of dietary conditions.

### Maternal OxLDL Immunization Protects Male Offspring Against IR under Severely Obesogenic Conditions

**Figure 6:**
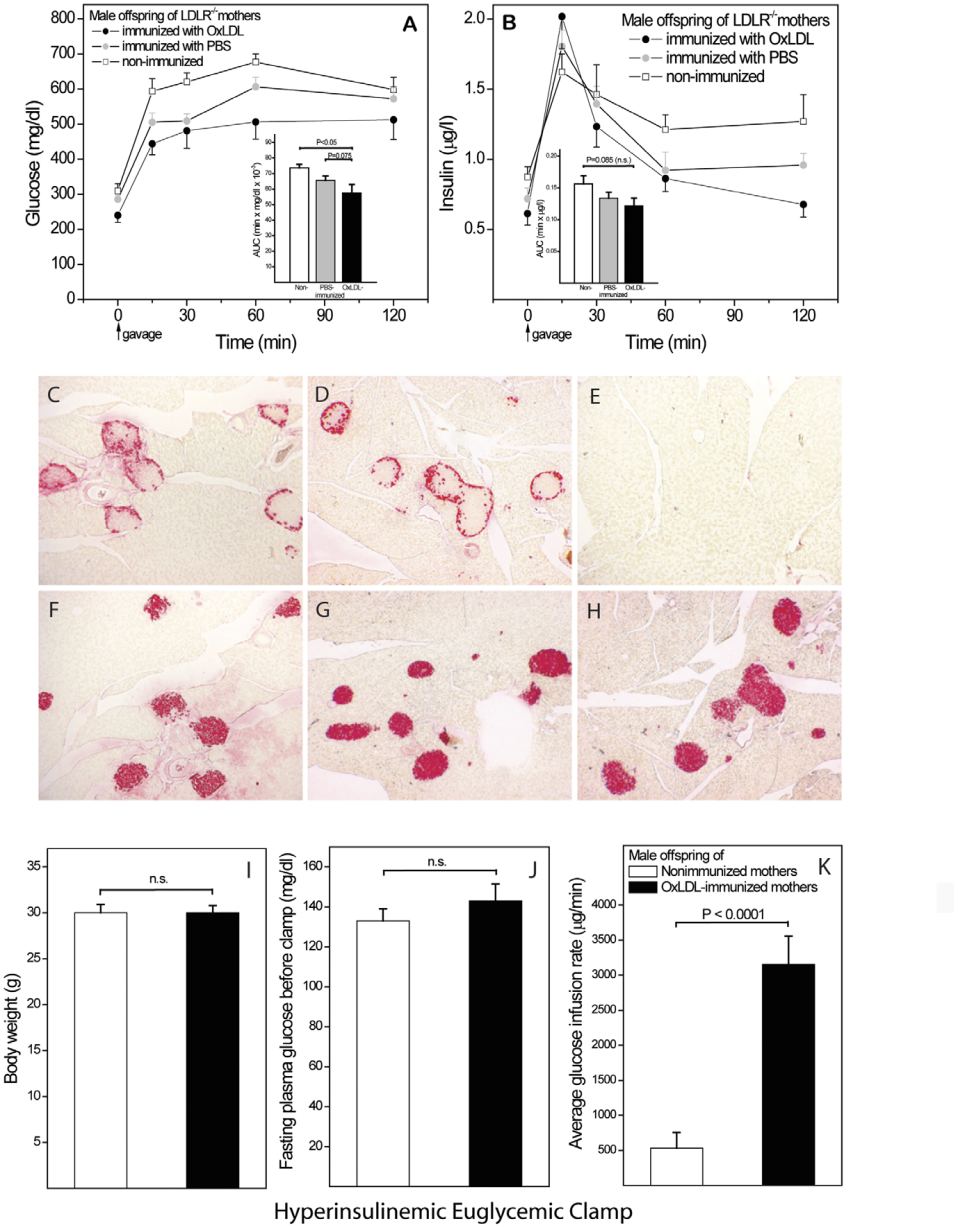
Protective effects of maternal OxLDL immunization in male offspring. (**A,B**) Glucose and insulin responses during an OGTT in male offspring of mothers immunized with OxLDL or PBS and non-immunized control mothers, after 28 weeks on 60% sucrose diet (n = 26). AUCs are shown as insets. (**C–H**) Pancreas immunohistochemistry of the same animals. Alpha cells identified by glucagon-staining in offspring of OxLDL-immunized (**C**) and control mothers (**D**). No-primary-antibody control (**E**). Beta cells staining for insulin in offspring of (**F**) OxLDL-immunized, (**G**) PBS-immunized and (**H**) control mothers (**I–K**)**.** An additional group of male offspring of OxLDL-immunized and control mothers was fed sucrose for 38 weeks and then subjected to hyperinsulinemic euglycemic clamp (n = 16). Body weights (I) and fasting plasma glucose levels (J) were not significantly different, whereas the amounts of glucose infused during the euglycemic phase was markedly greater in offspring of OxLDL-immunized mothers (K).

We then investigated the effects of maternal OxLDL immunization on IR in male offspring exposed to highly obesogenic conditions **(** **Figure 6** **)**. After 28 weeks on 60% sucrose diet, no effect of immunization on body weights were noted, but offspring of OxLDL-immunized mothers showed significantly better glucose responses than controls **(A)**. In contrast to directly immunized males (Fig. 5D,E), offspring insulin levels were only marginally greater in controls **(B)**. To assess whether part of the protective effect may be due to greater protection of beta cells in offspring of OxLDL-immunized mothers, pancreatic immunohistochemistry was performed. No differences in the number of glucagon-positive alpha cells **(C,D)** or insulin-positive beta cells **(F–H)** per section were detected. However, the beta cell/alpha cell ratio showed a clear trend of being higher in offspring of OxLDL-immunized mothers than in offspring of PBS-immunized and control mothers (1.72±0.14; 1.51±0.15 and 1.44±0.04, respectively; P = 0.054 OxLDL vs. control, n = 26). Thus, immunization may also convey some protection against beta cell deterioration.

To establish beyond doubt that OGTT results indicate a protective effect of maternal immunization against offspring IR, hyperinsulinemic euglycemic clamps were performed on two additional groups of male offspring of OxLDL-immunized and non-immunized mothers fed 60% sucrose for 38 weeks. Body weights **(I)** and fasting glucose levels **(J)** were similar in both groups, whereas the amount of glucose infused during the 30 min euglycemic period was markedly greater in offspring of OxLDL-immunized mothers **(K)**. Maternal OxLDL-immunization therefore protects against IR, in addition to any protection it may convey against adverse effects of the sucrose diet to beta cells.

### Maternal OxLDL Immunization Protects Female Offspring Against IR and Diabetes

**Figure 7:**
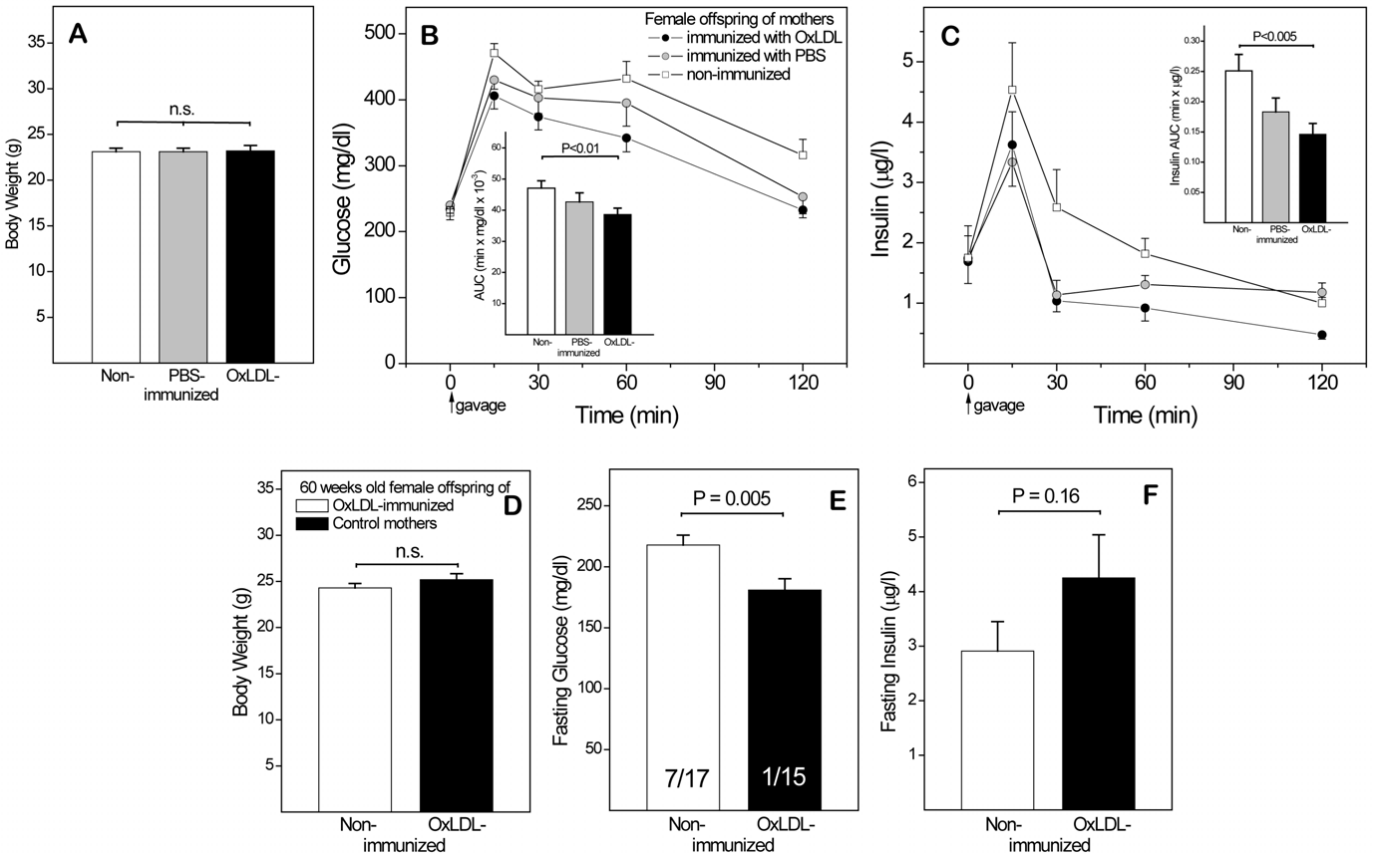
Protective effects of maternal OxLDL immunization in female offspring. (**A–C**) Female offspring of mothers immunized with OxLDL or PBS and non-immunized controls fed 60% sucrose diet for 24 weeks (n = 27). Body weights, glucose responses and insulin responses during an OGTT at this time are shown, together with respective AUCs (insets). (**D–F**) A second group of female offspring was reverted to regular chow for 19 weeks after insulin resistance had been induced by 36 weeks on the sucrose diet. Body weights, plasma glucose and insulin levels (after 6 hr. fasting) were determined at 60 weeks of age. Numbers of mice with glucose levels ≥230 mg/dl and total number in each group are indicated in the bars of panel E.

Female mice fed the 60% sucrose diet since weaning gained considerably less weight than males, both in absolute and relative terms. No significant effect of maternal immunization was evident in female subgroups subjected to OGTT after 12 and 18 weeks on diet (data not shown). After 24 weeks **(** **Figure 7** **)**, female offspring still weighed significantly less than males (**A)**, but glucose **(B)** and insulin **(C)** responses resembled those in male offspring (Figure 6), establishing that the protective effect of maternal OxLDL immunization is not gender-specific.

To follow the effects into old age without incurring *adverse* long-term effects of the diet, additional batches of female offspring were exposed to 36 weeks of sucrose diet followed by 19 weeks on regular chow. At age 59 weeks, body weighs **(D)** showed only a marginal increase, compared to 28 weeks (A), but fasting glucose levels were still significantly lower in offspring of OxLDL-immunized mothers than in offspring of controls **(E)**. Furthermore, 7 of 17 animals in the control group were hyperglycemic (fasting glucose ≥230 mg/dl), compared to only 1 of 15 in the OxLDL group (E, insets in bars). Insulin responses also deviated from those previously observed in IR mice, in so far as controls failed to generate sufficient insulin to maintain euglycemia. Together, these results indicate that maternal immunization not only protects offspring against IR, but also delays the transition to type 2 diabetes.

### Maternal OxLDL Immunization Influences Hepatic mRNA Expression and Increases the Activity of Antioxidant Enzymes in Offspring

Given that oxidative stress is prominently involved in atherogenesis and, putatively, also in IR, we then investigated effects of *in utero* programming on hepatic gene expression and activity of antioxidant enzymes in the liver and cardiac ventricle of offspring. Both parameters were measured in male offspring after 28 weeks on sucrose diet characterized in Fig. 6A,B. Comparison by qPCR array of hepatic mRNA expression of 84 genes known to influence oxidative stress or to be transcriptionally regulated by oxidative stress indicated 19 genes that were significantly changed >1.5-fold in offspring of OxLDL-immunized mothers, compared to controls (data not shown). These included superoxide dismutase (SOD) 1 and 2, glutathion peroxidase (GPx), peroxiredoxins 1, 3 and 4, NAD(P)H dehydrogenase, and thioredoxin reductase 1 and 3. An analogous array of 84 genes of interest for diabetes indicated only 4 genes that were significantly regulated by maternal immunization, albeit to a greater degree **(** **Table 1** **)**.

**Table 1:** Effect of maternal OxLDL immunization on hepatic expression of genes of relevance for diabetes.

Gene	Fold Regulation	P
Adrenergic receptor, alpha 1a	−1.76	0.02
Interleukin 10	−2.10	0.09
Peroxisome proliferator activated receptor alpha	−1.58	0.05
Solute carrier family 2 (facilitated glucose transporter), member 4	−2.83	0.04
Tribbles homolog 3	−1.99	0.04

mRNA expression of genes known to be regulated in diabetic conditions was assessed by commercial gene array in the liver of male offspring of OxLDL-immunized and control mice after 28 weeks on 60% sucrose diet (experiment #4). Only genes significantly regulated more than 1.5 fold are shown. IL-10 is included even though it failed to reach significance.

**Figure 8:**
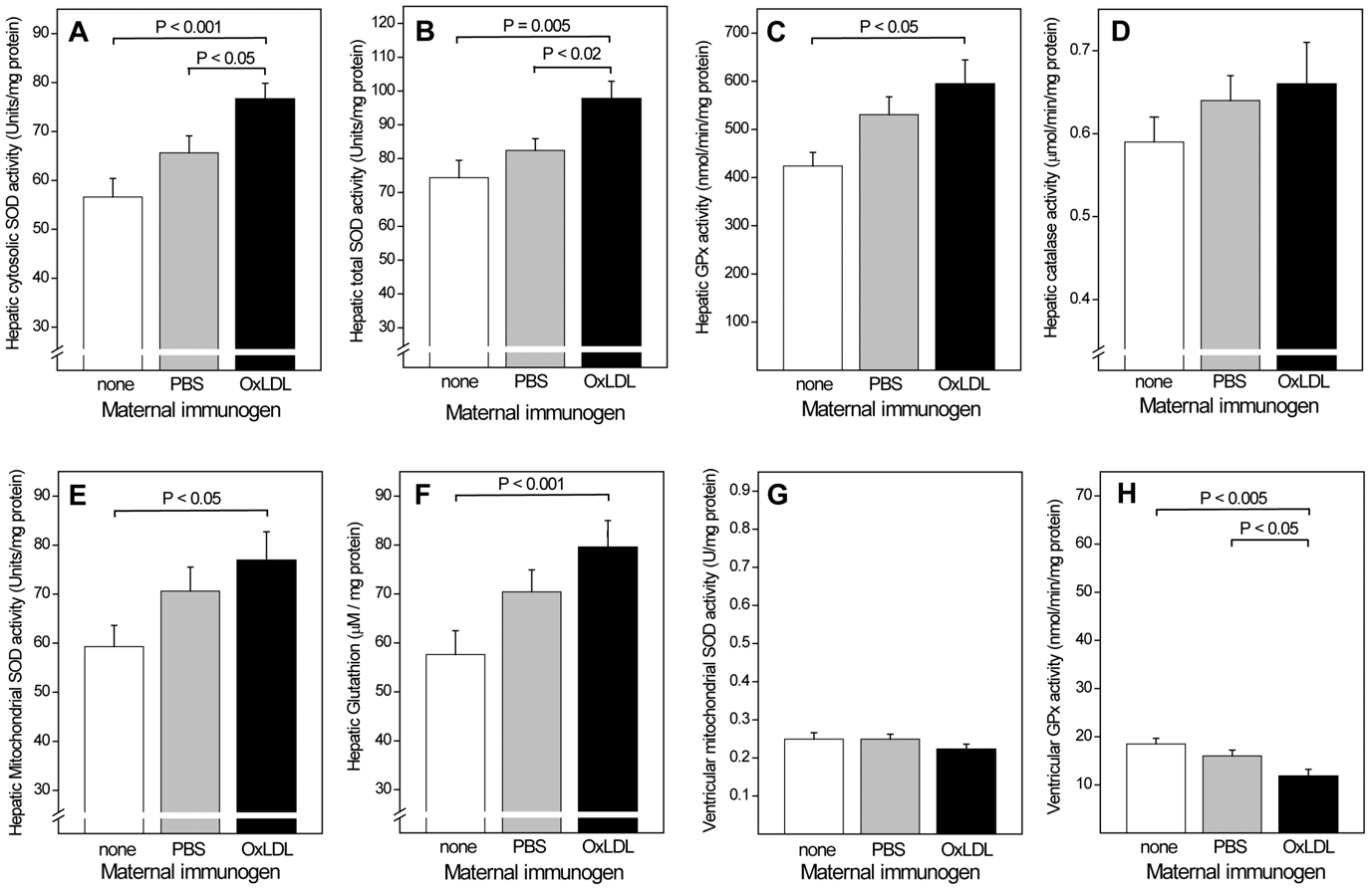
Effects of maternal OxLDL immunization on the activity of antioxidant enzymes and glutathion concentration in offspring. Hepatic activity of (A) cytosolic superoxide dismutase (SOD), (B) total SOD, (C) glutathione peroxidase (GPx), (D) catalase, and (E) mitochondrial SOD. (F) Hepatic glutathion concentration. Ventricular activities of mitochondrial SOD (G) and (H) GPx.

Hepatic activities of key antioxidant enzymes were consistently higher in offspring of OxLDL-immunized mothers than in controls **(** **Figure 8** **)**, with a 35% and 32% increase in cytosolic and total SOD (P<0.001 and P = 0.005, respectively) **(A,B),** a 29% increase in GPx (P = 0.05) **(C)**, and a trend towards an 11% increase in catalase **(D)**. Mitochondrial SOD activity, too, was significantly greater in offspring of OxLDL immunized mothers **(E)**. Higher hepatic glutathion (GSH) concentrations **(F)** and GSH/GSSG ratios (not shown) were also consistent with a better overall antioxidant protection of offspring of OxLDL-immunized mothers. Antioxidant enzyme activities in the ventricular part of the heart were much lower than in the liver and no differences were seen in the activities of cytosolic SOD, total SOD, catalase (data not shown) and mitochondrial SOD **(G)**. GPx activity was actually lower in offspring of immunized mice **(H).** This suggests that *in utero* programming is cell- or organ-selective.

## Discussion

The present study allows several conclusions. First, it shows that *in utero* programming by maternal hypercholesterolemia influences IR and diabetes. An influence of *in utero* conditions on diabetes has long been assumed, based on epidemiology, but the association was mainly with low birth weight and maternal factors impairing intrauterine growth [28]–[31]. Experimental evidence has linked maternal obesity with increased IR [32], but the only previous evidence for a role of maternal hypercholesterolemia was obtained in offspring of rat mothers fed high-fat diets both during pregnancy and lactation [17]. Diabetogenic *in utero* programming in our LDLR^−/−^ mice most likely resulted from the same factors causing atherogenic programming in this and other animal models, i.e. hypercholesterolemia and associated oxidative stress [1], [2], [6].

Second, and more importantly, the fact that maternal immunization prior to pregnancy reduces the susceptibility to IR and diabetes in offspring shows, in principle, that *in utero* programming of diabetic conditions can be inhibited by maternal immunomodulation, in analogy to the inhibition of atherogenic *in utero* programming. Notably, the protective effect was seen even in non-obese euglycemic females. To what extent the protective effect is due to the prevention of diabetogenic programming, as opposed to beneficial effects of immune programming resulting from maternal immunization [2], remains to be established. Similarly, we do not know the contribution of the increased hepatic activity of antioxidant enzymes observed in offspring of immunized mothers. It is possible that higher antioxidative activities are merely concomitant with a lower level of oxidative stress resulting from other factors. Theoretically, higher antioxidant enzyme activities could even be a consequence of, rather than a contributor to, better glucose homeostasis. We recently reported a broad decrease of plasma eicosanoid regulators of inflammation and immunity in offspring of OxLDL-immunized hypercholesterolemic NZW rabbits, for which the same caveat regarding causality applies [16]. Together however, the observation of altered antioxidant enzyme activities, altered plasma eicosanoids, and altered lymphocyte-dependent immunity suggests that the reduction of offspring diseases involves multiple mechanisms affecting inflammation and oxidative stress, rather than *in utero* programming of a few individual genes or gene products.

In contrast to the mechanisms by which *in utero* programming affects diseases, the direct effects of OxLDL immunization and the mechanisms by which they may protect immunized subject or affect *in utero* programming are known. Oxidation-specific epitopes are a prominent feature of atherosclerotic lesions and are enhanced in diabetes by interactions between oxidation and nonenzymatic glycation (“glycoxylation”) [33], [34]. OxLDL immunization greatly increases the titers of IgG and IgM antibodies binding to oxidatively modified lipoproteins, proteins and phospholipids. Removal from the circulation of immune complexes formed by such antibodies constitutes one of the mechanisms contributing to the reduction of atherogenesis in immunized animals [26]. Similar mechanisms are likely to protect the fetus against *in utero* programming by oxidized fatty acids and other mediators of maternal oxidative stress capable of crossing the placental barrier [2].

Third, the protective effect noted in both immunized animals and their offspring may provide new insights into the pathogenesis of IR and type 2 diabetes. The search for causes of IR has long focused on genetic defects in insulin receptors, insulin receptor ligands, and their downstream signaling, as well as on factors impairing insulin sensitivity that are typically increased in obesity, such as non-esterified fatty acids [35]. However, a prominent role of inflammation and an involvement of immune cells and their cytokines is now well established. Evidence for the destruction of pancreatic islet cells by IL-1 was first provided for type 1 diabetes [36], but in type 2 diabetes IL-1ß and other proinflammatory cytokines also disrupt insulin signaling, and IκB kinase ß links obesity-induced IR and inflammation through activation of NFκB [37], [38]. Conversely, several antinflammatory drugs improve insulin and glucose responses [38], [39]. IR is also attributed to increased activation of M1 macrophages in adipose tissue and a concomitant decrease of anti-inflammatory M2 macrophages [40], [41]. The question why macrophages should be activated in the apparent absence of autoimmune reactions was recently answered by the identification of the NLRP3 inflammasome as an instigator of obesity-induced inflammation and IR [42]. On the other hand, atherosclerosis research has established that macrophages are activated by hypercholesterolemia and OxLDL, and that they then further promote formation of reactive oxygen species and lipid peroxidation [43]. Furthermore, the formation of autoantibodies to oxidized LDL also constitutes a form of autoimmune response. Increased oxidative stress influences several modulators of insulin sensitivity regulated by oxidation-sensitive pathways, such as the NFkB, PPAR, and apoptotic pathways [44]. Interactions may also occur through the Ras–mitogen-activated protein kinase (MAPK) pathway that regulates nonmetabolic effects of insulin, such as cell survival [45], [46]. Oxidative stress has therefore previously been proposed to enhance IR and type 2 diabetes [47], but to date antioxidant interventions in insulin resistant or diabetic patients and animal models have not confirmed its causal role for IR. Elucidating the role of oxidative stress is further complicated by the fact that increased lipid peroxidation may both promote IR (e.g. by increasing TNFα and activating c-Jun kinase and IκB kinase-β) [35] and inhibit it by increasing insulin sensitivity (e.g. by downregulating proinflammatory cytokines). Inducible nitric oxide synthase, another oxidation-sensitive factor of importance for endothelial function, also affects IR [48]. Very recently, maternal protein deficiency was reported to reduce expression of antioxidant enzyme activity and increase oxidative stress in the pancreas of offspring [31]. IR in mice has also been proposed to be a cellular antioxidant defense mechanism associated with reduced mitochondrial SOD expression and prevented by mitochondrial SOD overexpression [49]. Our present observation of higher mitochondrial SOD activity in offspring of immunized mothers is consistent with these observations. The increased activity of antioxidant enzymes in the liver may therefore protect against IR. In view of the important role of immune mechanisms in IR, however, multiple interactions at the level of immune cells and cytokines can be envisioned to contribute to the protective effect of maternal immunization.

Our results raise the possibility that other maternal immunomodulation may also protect offspring against diabetic conditions. Clearly, offspring are not protected against the full spectrum of antigens the mother is immune to. In the case of maternal immunization with OxLDL, immune protection later in life may depend on natural post-natal boosting provided by the ubiquitous occurrence of the antigen and its increased formation by high-risk diets. However, there is no reason to believe that maternal immunization with diabetes-specific antigens may not also be protective, in particular if such immunity were deliberately enhanced after birth.

Potential caveats: Differences in the genetic background of various murine strains are known to greatly influence their susceptibility to inflammation and obesogenic or atherogenic diets. It is therefore important that consistent findings were obtained under a broad range of conditions ranging from euglycemia in females exclusively fed regular chow to extensive IR in highly obese sucrose-fed males. Another caveat is the difficulty to separate effects on IR from those on aging. Antioxidant protection decreases with age, whereas obesity, IR and diabetes tend to increase. We cannot exclude that some of the protective effects are due to effects of immunization on *in utero* programming of aging processes [30].

From a clinical perspective, the most important insights are that IR can be attenuated by immunization and that maternal immunomodulation can reduce diabetic conditions in their offspring. Immunization of prospective mothers before pregnancy, ideally before they develop hypercholesterolemia or obesity, avoids the risks of pharmaceutical interventions during pregnancy, but may effectively protect offspring against IR and atherosclerosis. Results also emphasize the importance of preventing gestational hypercholesterolemia and obesity by conventional approaches and the persistent benefits for offspring of brief maternal treatment.
